# Pediatric palliative care and end-of-life: a systematic review of economic health analyses

**DOI:** 10.1590/1984-0462/2022/40/2021002

**Published:** 2022-01-05

**Authors:** Denise Swei Lo, Noely Hein, Jaqueline Vilela Bulgareli

**Affiliations:** a Universidade de São Paulo, São Paulo, SP, Brazil.; b Universidade Federal de Uberlândia, Uberlândia, MG, Brazil.

**Keywords:** Systematic review, Palliative care, Hospice care, Health economics, Pediatrics, Revisão sistemática, Cuidados paliativos, Cuidados paliativos na terminalidade da vida, Economia da saúde, Pediatria

## Abstract

**Objective:**

To perform a systematic review of the health economic evidence on the care of children and adolescents with complex clinical conditions, comparing groups included and not included (control group) in palliative care at the end of life.

**Data source:**

The seven databases searched were PubMed, Embase, Web of Science, Cochrane Library, Virtual Health Library–Latin American and Caribbean Health Sciences Literature (VHL-LILACS), EBSCOhost, and Paediatric Economic Database Evaluation, following recommendations of the Preferred Reporting Items for Systematic Reviews and Meta-Analyses (PRISMA) Statement, from January 1979 to November 2020. The review included studies of patients under 18 years of age with complex clinical conditions that compared a palliative care group with a control group. The economic outcomes analyzed were length and place of stay at the end of life (home, hospice, ward, intensive care unit, emergency room), diagnostic and therapeutic procedures performed, and health-related costs. The exclusion criteria were: studies without a matched control group, conference/congress abstracts, letters to the editor, editorials, comments, qualitative studies, narrative reviews, studies with ten or fewer participants in each group, articles published in languages other than English, Portuguese, or Spanish.

**Data synthesis:**

Out of the 518 articles identified, 4 met the inclusion criteria. We found evidence of direct economic benefits, such as reduced health costs, indirect savings, and protection of patients from undergoing invasive procedures, surgeries, and costly therapies, which cause greater suffering at the end of life. Therefore, participating in a palliative care program saved financial and technological resources, besides increasing the frequency of deaths at home and improving the quality of life.

**Conclusions:**

Public and private policies to promote palliative care represent better efficiency when allocating available health care resources.

## Resumo

**Objetivo:**

Realizar revisão sistemática das evidências de economia da saúde no cuidado de crianças e adolescentes com condições clínicas complexas, comparando no fim de vida o grupo inserido em cuidados paliativos com o grupo não inserido (grupo controle).

**Fontes de dados:**

As sete bases de dados pesquisadas foram PubMed, Embase, Web of Science, Cochrane Library, Biblioteca Virtual da Saúde-Literatura Latino-Americana e do Caribe em Ciências da Saúde (BVS-LILACS), EBSCOhost e Paediatric Economic Database Evaluation, seguindo as recomendações do Preferred Reporting Items for Systematic Reviews and Meta-Analyses (PRISMA) Statement, de janeiro/1979 a novembro/2020. A revisão incluiu estudos com pacientes com condições clínicas complexas, idade inferior a 18 anos, comparativos de um grupo inserido em cuidados paliativos com um grupo controle não inserido em cuidados paliativos. Os desfechos econômicos analisados foram tempo e local de permanência no fim de vida (casa, *hospice*, enfermaria, unidade de terapia intensiva, pronto-socorro), procedimentos diagnósticos e terapêuticos realizados e custos relacionados aos cuidados de saúde. Os critérios de exclusão foram: estudos sem grupo controle pareados, resumos de conferências/congressos, cartas ao editor, editoriais, comentários, estudos qualitativos, revisões narrativas, estudos com dez ou menos participantes, artigos publicados em outras línguas além de inglês, português e espanhol.

**Síntese dos dados:**

Do total de 518 artigos identificados, quatro preencheram os critérios de inclusão. Houve evidências de benefícios econômicos diretos de redução de custos monetários relacionados à saúde e também indiretos, de economia e proteção ao paciente de procedimentos invasivos, cirurgias e terapias onerosas, que geram maior sofrimento no fim de vida. Portanto, estar inserido em um programa de cuidados paliativos promoveu economia de recursos financeiros e tecnológicos, além de ter possibilitado maior frequência de óbitos no domicílio e maior qualidade de vida.

**Conclusões:**

Políticas públicas e privadas para promover cuidados paliativos representam melhor eficiência na alocação dos recursos disponíveis para cuidados em saúde.

## INTRODUCTION

Pediatric palliative care is an active approach that improves the quality of life of patients and families who face problems associated with life-threatening diseases.^1^ It prevents and alleviates suffering through early identification, evaluation, and treatment of pain and other conditions, whether physical, psychosocial, or spiritual. The multidisciplinary approach is recommended and should start as soon as the disease is diagnosed, as it does not exclude the active treatment of the clinical condition. Therefore, palliative care promotes the quality of life and considers death a natural process, which should not be abbreviated or extended at the expense of futile procedures and suffering.^2^

Complex clinical conditions eligible for palliative care, including congenital malformations or deformities, chromosomal abnormalities, and conditions that originated in the neonatal period (due to prematurity and low weight), are the main causes of infant mortality in countries such as the United States of America (USA)^3^ and Brazil.^4^ These causes corresponded to 38% of the 21,498 deaths of children under 1 year of age in the USA in 2018, according to data from the Centers for Disease Control and Prevention (CDC).^3^ In Brazil, official data from the Technology Department of the public health system (*Departamento de Informática do Sistema Único de Saúde* — DATASUS) reveals that 20,738 (57.8%) out of 35,684 deaths of children under 1 year of age were due to diseases originating in the neonatal period and 8,313 (23.2%) to congenital malformations, deformities, and chromosomal abnormalities in 2018.^4^

The literature has ample scientific evidence that providing palliative care improves the quality of life of patients and families facing a condition that shortens life.^5,6,7^ Bioethical principles of beneficence and non-maleficence (ethical obligation to maximize benefit and minimize harm) should be followed for all ages, ethnicities, genders, and social classes.^8,9^ The Brazilian Ministry of Health, in its Resolution no. 41 of October 31, 2018, standardized palliative care as part of the integrated continuous care offered by the health care system;^10,11^ however, few measures have been taken, and scarce resources have been provided for the effective implementation of this standard, especially for pediatric patients.

The financial costs of health technologies can be quite high for patients with complex clinical conditions at the end of life. Thus, providing palliative care is an ethical, legal, humanitarian, social, and also necessary principle in the field of health economics. Optimizing health actions is essential, namely, distributing the available resources to ensure the population’s best possible health care and health status, considering the limited means and resources.

This study aimed to perform a systematic review of health economics — in its different dimensions — related to the treatment of children and adolescents with complex clinical conditions, comparing groups included and not included in palliative care. Our objective was to provide technical-scientific support and help improve the administration of available resources and strategic decision-making in health services management.

## METHOD

The method used in this review follows the Preferred Reporting Items for Systematic Reviews and Meta-Analyses (PRISMA) Protocol.^12^ This protocol was duly registered in the International Prospective Register of Systematic Reviews of the National Institute for Health Research (PROSPERO — CRD 42020190957) under the title “Pediatric palliative care and end-of-life: a systematic review of health economic analyses”.

The PICOS acronym of PRISMA^12^ used in this systematic review was individualized as inclusion criteria:

- Participants: population of patients under 18 years of age with complex clinical conditions at the end of life (defined as the last 6 months of life);^13,14^
- Intervention: group participating in a palliative care program;
- Comparison/context: control group not participating in end-of-life palliative care programs;
- Outcomes: length and place of stay at the end of life (home, hospice, ward, intensive care unit, emergency room), diagnostic and therapeutic procedures performed, and health-related costs.
- Study design: prospective/retrospective observational cohorts with a control group and case-control studies.

The exclusion criteria were: studies without a matched control group, conference/congress abstracts, letters to the editor, editorials, comments, qualitative studies, narrative reviews, studies with ten or fewer participants in each group, articles published in languages other than English, Portuguese, or Spanish.

Librarians specialized in the systematic review process contributed to this study to construct the best full search syntax for the available databases from January 1979 to November 16, 2020. The databases searched were: PubMed, Embase, Web of Science, Cochrane Library, Virtual Health Library–Latin American and Caribbean Health Sciences Literature (VHL-LILACS), EBSCOhost (Academic Search Premier/CINAHL/EconLit/Medical Literature Analysis and Retrieval System Online — MEDLINE/Public Administration Abstracts), and Paediatric Economic Database Evaluation (PEDE). Google Scholar was used for the complementary search of relevant references of the main articles.

Descriptors were based on the Medical Subject Headings (MeSH), keywords, and entry terms. The terms for the population were: Pediatric* OR Paediatric* OR Child* OR Infant* OR Newborn* OR Adolescent*. The terms for the intervention were: Hospice and Palliative Care Nursing OR Palliative Medicine OR Palliative Care OR Hospice Care OR hospice*. The terms for the end-of-life context were: Terminal Care OR End-of-life* OR End of life* OR Critical illness. The terms for economic outcomes were: Cost* OR Economic* OR Health expenditures OR Technology, high-cost OR Technology High-cost. The final syntax was the combination (AND) of the four groups cited (result table available with the corresponding author).

Using the spreadsheet of articles found, two researchers (first and second authors) independently evaluated the possible inclusion of these studies based on title and abstract. Duplicate articles were removed, and cited articles related to the review theme were included. In the next step, the articles were read in full, also independently, to select which ones to include in the systematic review. In case of disagreement, the third author was consulted to reach a consensus. The results of the articles selected were presented as tables describing the characteristics of the studies and the main economic effects found.

We assessed the methodological quality with the Checklist for Systematic Reviews and Research Syntheses, developed by the Joanna Briggs Institute Critical Appraisal tools for use in JBI systematic reviews.^15^ This instrument was chosen because it was adequate for the synthesis of economic issues, especially in studies such as the one proposed, which cannot be conducted through randomized clinical trials. Given the heterogeneity of the data evaluated, the statistical method of data synthesis was performed narratively, as these data cannot be combined for a meta-analysis.

## RESULTS

**Figure 1.**
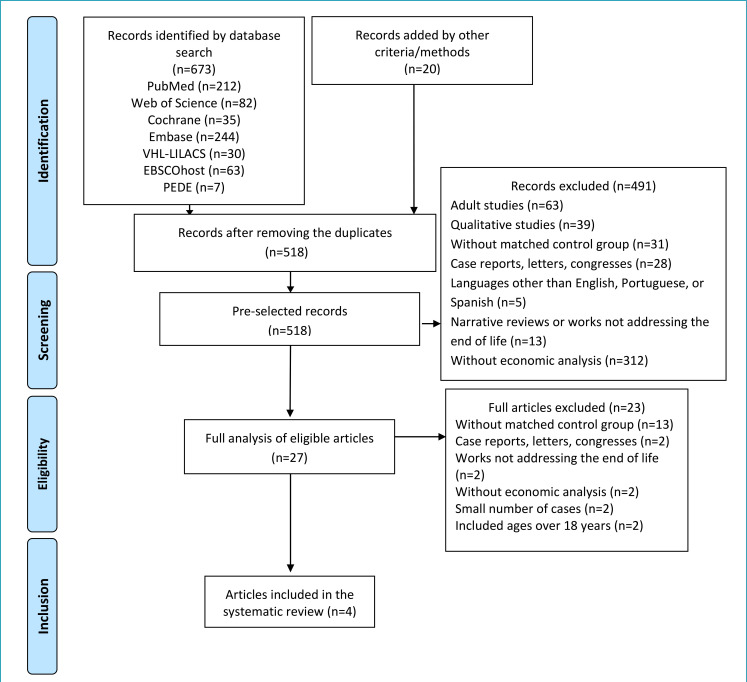
PRISMA flow diagram of the systematic review.

VHL-LILACS: Virtual Health Library–Latin American and Caribbean Health Sciences Literature; EBSCOhost: Academic Search Premier/CINAHL/EconLit/Medical Literature Analysis and Retrieval System Online – MEDLINE/Public Administration Abstracts; PEDE: Paediatric Economic Database Evaluation; PRISMA: Preferred Reporting Items for Systematic Reviews and Meta-Analyses.

Figure 1 depicts the PRISMA flow diagram used to evaluate and select the articles and the reasons for excluding them. Out of the 518 articles found after removing duplicates, only 4 met the final requirements for inclusion in this systematic review. The main reasons for exclusion were: theme not related to economic analysis (n=314); adult studies/inclusion of ages over 18 years (n=65); studies without a matched control group (n=44); qualitative studies (n=39); case reports, letters, or congress abstracts (n=30); narrative reviews or works not addressing the end of life (n=15); languages other than English, Portuguese, or Spanish (n=5); studies with 10 or fewer participants in each group (n=2).

Table 1 presents the characteristics of the studies included in this systematic review.^16–19^ Only investigations carried out in countries with a high human development index^20^ met the inclusion criteria; among them, two were retrospective cohorts, and two were retrospective case-control studies. Comparative economic outcomes ranged from the cost of health-related expenses to indirect costs, such as differences in emergency room use, hospital admissions and length of stay, stay in intensive care units, and diagnostic and therapeutic procedures performed.

**Table 1.** Characteristics of the studies included in the systematic review.

Main author	Study period	Country	Case description	Age (years)	Method	Patients (n)		Outcome description
						Palliative care	Control group	
Smith et al.^16^	2010 to 2012	United States of America	The 10% most costly inpatients in 2010	0–18	Retrospective cohort	86	816	Comparison between groups regarding costs and days of hospitalization, daily cost per patient, daily costs before and after inclusion in palliative care programs.
Chong et al.^17^	2012 to 2015	Singapore	Children and adolescents with complex clinical conditions (more than 40% with neoplasms)	0–18	Retrospective case-control	71	67	Evaluation of the number and length of hospitalizations, visits to the emergency department, and medical expenses in the last year of life
Keele et al.^18^	2001 to 2011	United States of America	Deaths recorded in the Pediatric Health Information System database, with at least five days of hospitalization	0–18	Retrospective cohort	919	23,423	Evaluation of the median days of hospitalization, number of invasive procedures, stay in the intensive care unit, and hospital expenses
Pierucci et al.^19^	1994 to 1997	United States of America	Deaths of children admitted to a tertiary hospital	0–1	Retrospective case-control	25	123	Assessment of the place of death and medical procedures performed

Table 2 summarizes the outcomes found and the main results. These economic outcomes varied according to survival time, as demonstrated in the study by Smith et al.^16^ Considering the 10% most costly inpatients of the Primary Children’s Hospital (Utah, USA) in 2010, the authors analyzed economic aspects comparing the palliative care group and the control group. They stratified three groups per survival time:

- Group 1: patients who died in 2010 or up to 10 days after hospital discharge;
- Group 2: patients who died after 11 to 730 days;
- Group 3: patients with survival above 730 days (therefore, outside the end-of-life period).

**Table 2.** Economic effects of pediatric palliative care versus conventional care.

Main author	Outcome summary	Description of the main results
Smith et al.^16^	The group that received palliative care had lower hospital expenses in the two years before death, while the survivors of this group had higher expenses. When adjusted for disease complexity, expenses were similar.	The palliative care group had more complex diseases. Economic results varied according to survival time. Hospitalization costs of patients who received palliative care and died in the following two years were lower than those of the control group, with no significant difference in the daily cost of hospitalization and admission to pediatric intensive care. Inclusion in the palliative care program reduced the mean daily cost from US$ 4,732 to US$ 3,625 (p<0.001).
Chong et al.^17^	Comparison between the palliative care group and the control group showed a significant decrease in hospitalizations and medical costs in the last year of life, particularly in the last month.	In the last year of life, compared to controls, patients in the palliative care group stayed more days at home than in hospital (OR 52.3; 95%CI 25.44−79.17), and 70% had lower medical expenses. Costs in the last month of life decreased by 87% in the palliative care group. The mean age in years of the palliative care group was significantly higher than that of controls (12.2 vs. 6.3). The quality of life of patients and family members improved after inclusion in the palliative care program.
Keele et al.^18^	The palliative care group had a higher median age and different complex clinical conditions than the control group. Comparison between the groups revealed fewer days of hospitalization, fewer invasive procedures, fewer deaths in the intensive care unit, and lower hospital expenses in the palliative care group.	The group receiving palliative care had lower median days of hospitalization than the control group (17 vs. 21), as well as reduced daily costs (US$ 9,348 vs. US$ 11,806), underwent fewer invasive procedures, and presented fewer deaths in the intensive care unit (60 vs. 80%). A significant difference was found concerning the higher age of the palliative care group. Children under 30 days of life corresponded to 41% of deaths, and only 2% of them received palliative care.
Pierucci et al.^19^	Infants who received palliative care were submitted to fewer diagnostic and therapeutic procedures and stayed fewer days in the intensive care unit than the control group.	Infants in palliative care showed significant reductions compared to controls, with a lower infusion of blood products (36 vs. 63%), central line insertion (64 vs. 92%), endotracheal intubation (60 vs. 94%), use of feeding tubes (64 vs. 95%), and X-ray examinations (40 vs. 89%). A significant difference was identified in the last 48 hours of life, with 44% of infants in the palliative care group without blood tests versus 7% in the control group. The use of vasopressors was limited to 56% of the palliative care group against 13% of the control group.

OR: odds ratio adjusted for age, length of stay, and diagnostic category; 95%CI: 95% confidence interval.

Comparing only absolute costs, the palliative care group had higher hospitalization costs than the control group (total annual mean of 2010=US$ 245,214 vs. US$ 231,072); however, the palliative care group was significantly more complex than the control group. Patients who received palliative care were older, more dependent on health technologies, had more admissions to intensive care units, more complex clinical conditions, and greater association with deaths in 2010. Patients in the palliative care group who died in 2011 and 2012 (Group 2) presented lower hospital costs and length of stay than the control group. Therefore, the study evidences the importance of adjusting baseline factors and time to death when comparing the economic aspects of the two groups.

Chong et al.^17^ described the palliative care program developed in Singapore — Star Paediatric Advanced Life Support —, which was clearly cost-effective compared to the control group, saving 70% of costs in the last year of life and 87% in the last month of life. The study by Keele et al.^18^ was based on mortality data from the Pediatric Health Information System, which includes more than 40 US hospitals. They assessed deaths of individuals under 18 years of age, with at least 5 days of hospitalization and disease diagnosis codes eligible for palliative care. Only 4% of a total of 24,342 cases received palliative care. The research demonstrated that participating in the palliative care group reduced a series of procedures, such as mechanical ventilation, invasive monitoring, and futile end-of-life therapies, including hemodialysis, transfusions, cardioversion, surgeries, and total parenteral nutrition. In addition, palliative care reduced hospitalizations and deaths in intensive care units. These findings were very similar to those previously described by Pierucci et al.^19^ for children who died before one year of life, indicating a significant decrease in invasive procedures and greater social and spiritual support for families.

This systematic review focused on synthesizing evidence relevant to health economics in palliative care. However, we emphasize that the articles included also report benefits such as fewer pain symptoms and better quality of life for patients in palliative care and their families.^17^ Other care aspects included the greater administration of medications related to symptom control in the palliative care group and/or the provision of support and comfort measures for patients and their families.^19^

## DISCUSSION

The synthesis of this systematic review revealed evidence of health economics when palliative care is provided at the end of life, both through direct measures to reduce health-related costs and indirect actions aimed at lowering costs and at cost-benefit, cost-effectiveness, and cost-utility. Economic benefits are more significant closer to death; however, we underline that palliative care should not be provided only in the final stage of life, but at the moment a life-threatening clinical condition is diagnosed, and it should continue throughout all stages of the disease until the post-mourning period.

The multidisciplinary approach recommended in palliative care programs seeks to early promote a better quality of life, assisting in physical comfort, as well as emotional, social, and spiritual support for patients and families. The holistic concept of palliative care also protects the patient from undergoing invasive procedures, surgeries, and therapies that are futile, costly, have few benefits, and inflict greater suffering at the end of life, thus saving financial and technological resources and increasing the frequency of deaths at home. This systematic review provides technical-scientific support for health managers to actively implement these programs, as they also save often scarce financial and technological resources.

Health economic studies on palliative care might differ according to survival time since curative treatment of reversible clinical conditions should be administered when these costly procedures benefit the patient’s quality of life. The challenge of predicting life expectancy is relevant when planning clinical decisions. Nonetheless, the scientific literature has not reached a consensus on the concept of end-of-life duration.^13,14^Smith et al.^16^ reported that patients in the palliative care group with less than two years of survival had significantly lower hospitalization costs than those not receiving palliative care. On the other hand, patients with complex clinical conditions, which often lead to higher health expenses, and over two years of survival — therefore, outside the end-of-life period — usually presented inverse results as to hospital costs.

The Worldwide Hospice Palliative Care Alliance (WHPCA), together with the World Health Organization (WHO), published an update to its Global Atlas of Palliative Care in 2020.^2^ In this document, the WHO states that palliative care is a human right. However, estimates indicate that 56.8 million people need palliative care every year, 25.7 million of them at the end of their lives. They consist mainly of adults over 50 years of age (67.1%), and at least 7% are children. Most adults and children (54.2%) need this support in their last year of life. The vast majority (>97%) of these children under the age of 19 live in underdeveloped and emerging countries.

Of note, all studies included in this review were carried out in countries with a high human development index,^20^ such as the USA and Singapore. The USA presented the highest category of palliative care development and integration (4b). Countries in this category have public and educational policies on palliative care, different services integrated within the community, unrestricted availability of strong painkillers such as morphine, as well as recognition from society and health professionals as to the positive impact of palliative care. Only 30 countries are in category 4b, representing 14.2% of the world’s population. Singapore was categorized as 4a, that is, a preliminary stage of palliative care integration into the health care system, the existence of a national palliative care association, with public policies and strategies of palliative care under development. Category 4a comprises 21 countries and 27.6% of the world’s population. Next is Brazil, in category 3b of WHPCA,^2^ which encompasses 22 countries and 5.7% of the world’s population.

Unfortunately, few papers met the inclusion criteria of this systematic review. The economic study of palliative care presents limitations for several reasons. First, these studies are retrospective due to the ethical impossibility of carrying out prospective designs. A wide range of evidence demonstrates the benefits of palliative care for the quality of life of patients and families in the face of complex life-threatening clinical conditions.^5–7^ Therefore, palliative care should be provided whenever indicated, except when the patient or their legal guardian refuses.

Furthermore, the palliative care group was not similar to the control group in all studies.^16–19^ The allocation of more severe patients in the palliative care group presents a natural clinical bias, given the clear medical perception of the impossibility of healing and of the end of life. As a result, the palliative care group often consists of patients with more complex clinical conditions than the control group, predisposing them to be inherently more expensive. Thus, comparative cost studies should be adjusted for the clinical complexity of patients.

Moreover, the end-of-life period is not clearly defined. Economic assessments may vary according to survival time. Scientific evidence reinforces that promoting palliative care reduces hospitalization costs^16–18^ and futile invasive procedures,^18,19^ in addition to increasing physical,^17^ emotional, and social support^17,19^ the closer the death.

Lastly, palliative care can be provided in different ways. The costs of this program differ based on the type of service offered, the number of professionals involved, the technology used, the public policies in the region, as well as the place where it will be provided (outpatient clinic, home, hospices, hospitalization units).

This systematic review of health economics in pediatric end-of-life palliative care demonstrated the need to elaborate effective public and private policies that promote palliative care programs. Palliative care measures should foster the inclusion of palliative care disciplines in undergraduate and specialization courses, as well as continuing education, for health professionals in different areas; encourage and promote multidisciplinary work; disseminate information and offer access to palliative care programs for society; provide access to drugs that control the patients’ symptoms; and offer the necessary and proper support for the patient so they can remain at home or at the hospice at the end of life.^2,21^ Palliative care optimizes health actions, in addition to being an ethical, legal, humanitarian, and social principle. This issue is urgent and relevant in the management of both public and private resources.
